# Using behavioural science for better health

**DOI:** 10.2471/BLT.21.287387

**Published:** 2021-11-01

**Authors:** Tedros Adhanom Ghebreyesus

**Affiliations:** a World Health Organization, Avenue Appia 20, 1211 Geneva 27, Switzerland.

Each year, two thirds of deaths globally are caused by noncommunicable diseases that result from genetic, physiological, environmental and behavioural factors.^1^ Behaviour affects health. For example, many risk factors causing noncommunicable diseases are behaviourally driven, such as smoking and sedentary lifestyle. The misuse of antibiotics in human health care is a main but avoidable driver of antimicrobial resistance.^2^ Hand hygiene is one of the most effective measures to stop infection spread but people often fail to keep adequate hand hygiene, even where water and soap are available.^3^ We should resist the temptation to conclude that people have insufficient knowledge or awareness of the importance of such matters: these are behavioural issues, and most if not all health challenges involve the behaviour of individuals.

By behavioural elements, we mean a health problem or outcome that can be determined by a person or community’s specific health-related actions. Such behaviours play a fundamental role in protecting us from health emergencies but also in accessing services and helping us enjoy better health.

To address global and local health challenges and threats, and achieve the sustainable development goals, the United Nations family needs to understand how and why people and communities behave in their respective contexts.^4^ As the global public health leader, the World Health Organization (WHO) cannot achieve its ambitious goal of transforming global health and the health of more than 7 billion people^5^ without a clear understanding of people’s health-related behaviours. We have therefore established a behavioural science unit at WHO and dedicate this theme issue of the *Bulletin of the World Health Organization* to this field. 

Human behaviours and decision-making are complex and driven by cognitive, social and environmental factors.^6^ Behavioural science is the evidence-based study of how people behave and why, and can help us anticipate people’s response to health programmes and policies.^4^ In the last decade, the adoption of behavioural science has contributed to the success of several public policies and programmes in a variety of fields such as consumer protection, education, energy, environment and taxation, including in health.^7^^,^^8^

However, behavioural science is not yet an integral part of daily practice for most public health work and it remains an underutilized resource. Barriers to the adoption of behavioural theories and approaches include practical ones such as lack of capacity, funds and time.^9^ Moreover, policy-makers and technical managers still lack confidence in the impact of these interventions or in the sustainability^10^ and replicability of insights and changes obtained.^11^

These barriers have decelerated the use of behavioural science in public health policy. More research, investment and stronger collaboration across sectors is needed to better address these barriers. While an encouraging increase in behavioural research has taken place, its application to global health remains limited and often driven by high-income country research agendas.^12^

As members of the broader public health community, we need to get better at listening and observing people and their needs. Doing so requires rigorous collection of behavioural data to understand, for example, how and why our minds take shortcuts, also called heuristics, when information is too much or unclear, or when the right choice is too hard to make.

We also need more information on how people around us influence our decisions, and on how to design environments, services, products and solutions that support, rather than block, behaviours that improve health.

Using behavioural insights is ultimately an act of humility: it requires the community of experts and policy-makers to test each other’s expert knowledge, biases and preferences, and to gather and use behavioural evidence on health-related decisions that all of us, as individuals, make on any given day.

This theme issue of the *Bulletin* on behavioural sciences for better health provides examples of the work of multidisciplinary teams across the world who have partnered to design interventions that have contributed to improve people’s health. These examples should encourage all those involved in public health to work more systematically in the same direction. Ensuring behaviourally informed strategies, policies and programmes – as opposed to siloed behavioural interventions – is essential to achieving and sustaining better health for all.
